# A stem cell medium containing neural stimulating factor induces a pancreatic cancer stem-like cell-enriched population

**DOI:** 10.3892/ijo.2014.2603

**Published:** 2014-08-14

**Authors:** YUSAKU WATANABE, KIYOSHI YOSHIMURA, KOICHI YOSHIKAWA, RYOICHI TSUNEDOMI, YOSHITARO SHINDO, SOU MATSUKUMA, NORIKO MAEDA, SHINSUKE KANEKIYO, NOBUAKI SUZUKI, ATSUO KURAMASU, KOUHEI SONODA, KOJI TAMADA, SEI KOBAYASHI, HIDEYUKI SAYA, SHOICHI HAZAMA, MASAAKI OKA

**Affiliations:** 1 Department of Digestive Surgery and Surgical Oncology, Yamaguchi University School of Medicine, Ube, Yamaguchi 755-8505, Japan; 2 Department of Neurosurgery, Yamaguchi University School of Medicine, Ube, Yamaguchi 755-8505, Japan; 3 Department of Molecular Pharmacology, Yamaguchi University School of Medicine, Ube, Yamaguchi 755-8505, Japan; 4 Department of Ophthalmology, Yamaguchi University School of Medicine, Ube, Yamaguchi 755-8505, Japan; 5 Department of Immunology and Cell Signaling Analysis, Yamaguchi University School of Medicine, Ube, Yamaguchi 755-8505, Japan; 6 Department of Molecular Physiology and Medical Bioregulation, Yamaguchi University School of Medicine, Ube, Yamaguchi 755-8505, Japan; 7 Division of Gene Regulation, Institute for Advanced Medical Research, Graduate School of Medicine, Keio University, Shinjuku-ku, Tokyo 160-8582, Japan

**Keywords:** cancer stem cells, cell culture method, pancreatic cancer

## Abstract

Cancer stem cells (CSCs) have been studied for their self-renewal capacity and pluripotency, as well as their resistance to anticancer therapy and their ability to metastasize to distant organs. CSCs are difficult to study because their population is quite low in tumor specimens. To overcome this problem, we established a culture method to induce a pancreatic cancer stem-like cell (P-CSLC)-enriched population from human pancreatic cancer cell lines. Human pancreatic cancer cell lines established at our department were cultured in CSC-inducing media containing epidermal growth factor (EGF), basic fibroblast growth factor (bFGF), leukemia inhibitory factor (LIF), neural cell survivor factor-1 (NSF-1), and N-acetylcysteine. Sphere cells were obtained and then transferred to a laminin-coated dish and cultured for approximately two months. The surface markers, gene expression, aldehyde dehydrogenase (ALDH) activity, cell cycle, and tumorigenicity of these induced cells were examined for their stem cell-like characteristics. The population of these induced cells expanded within a few months. The ratio of CD24high, CD44high, epithelial specific antigen (ESA) high, and CD44variant (CD44v) high cells in the induced cells was greatly enriched. The induced cells stayed in the G0/G1 phase and demonstrated mesenchymal and stemness properties. The induced cells had high tumorigenic potential. Thus, we established a culture method to induce a P-CSLCenriched population from human pancreatic cancer cell lines. The CSLC population was enriched approximately 100-fold with this method. Our culture method may contribute to the precise analysis of CSCs and thus support the establishment of CSC-targeting therapy.

## Introduction

CSCs have been studied in terms of their self-renewal capability and pluripotency, as well as their resistance to anticancer therapy and ability to metastasize to distant organs (1,2). Conventional chemotherapies and radiation therapies were initially developed targeting the cancer-cell population. However, these treatments have no efficacy against CSCs, which have been shown to be resistant to standard chemotherapeutic agents (3–5). Pancreatic cancer is the 5th most common cause of cancer death in Japan (Center for Cancer Control and Information Services, National Cancer Center, Japan). The overall 5-year survival rate worldwide is <10% (6). The prognosis for pancreatic cancer patients with hepatic metastases is dismal because these patients cannot have radical surgery. Thus, novel and effective treatments against pancreatic CSCs are greatly needed. CSCs can be identified and isolated by different methodologies, including isolation by CSC-specific cell surface marker expression, detection of side population phenotype by Hoechst 33342 exclusion, and assessment of their ability to grow as floating spheres (7–13). However, the population of CSCs in tumor specimens is quite low; therefore, it is difficult to obtain purified CSCs in adequate numbers for effective study. To overcome this problem, we established a culture method to induce a P-CSLC-enriched population from human pancreatic cancer cell lines. In long-term culture, these induced cells maintained their stem-like phenotype as characterized by: i) the ability to survive under harsh conditions created by the media without serum and with EGF, bFGF, LIF, and NSF-1, in which non-stem-like cancer cells are not able to survive; ii) sphere-shaped morphology; and iii) longer survival in laminin-coated dishes. This method is stable and durable and will support the establishment of CSC-targeting therapy by consistently providing abundant CSCs.

## Materials and methods

### Culture of human pancreatic cancer cell lines

The human cancer cell lines used in the experimental study were pancreatic cancer cell lines YPK2 and YPK5, which were established in our department (14). Cell lines were maintained in DMEM-F12 (Sigma-Aldrich, Tokyo, Japan) containing 10% heat-inactivated FBS (Life Technologies, Tokyo, Japan) at 37°C in 5% CO_2_.

### Induction and culture of CSLC-enriched population

Cells were initially cultured in serum-free medium which is based on neural stem cell medium. The basal medium for the sphere induction is DMEM-F12 supplemented with 10 mM HEPES (Sigma-Aldrich), 1× antibiotic antimycotic solution (Sigma-Aldrich), 0.6% glucose (Sigma-Aldrich), 1 mg/ml transferrin, 250 μg/ml insulin (Sigma-Aldrich), 0.6 mM putrescine (Sigma-Aldrich), 0.3 μM sodium selenite (Sigma-Aldrich), and 0.2 μM progesterone (Sigma-Aldrich). Complete sphere induction medium was prepared by adding 2 μg/ml heparin (Sigma-Aldrich), 20 ng/ml EGF (Sigma-Aldrich), 20 ng/ml bFGF (Merck Millipore, Tokyo, Japan), 10 ng/ml LIF (Merck Millipore), 1/50 Vol NSF-1 (Lonza, Tokyo, Japan), and 60 μg/ml N-acetyl-L-cysteine (Sigma-Aldrich). Upon the formation of spheres, the sphere cells (YPK2-Sp and YPK5-Sp) were collected. YPK2-Sp or YPK5-Sp were then transferred to a laminin-coated dish with the sphere culture medium containing 20 μl/ml B27 supplement (Life Technologies), 1× antibiotic antimycotic solution, 75 μg/ml BSA (Sigma-Aldrich), 10 ng/ml EGF, and 10 ng/ml bFGF. Medium was renewed by a 50% change every 7 days. Cells became attached and gradually divided and increased in number (YPK2-Lm and YPK5-Lm).

### Flow cytometry analysis and sorting

Dissociated cells were counted and transferred to a 5-ml tube, washed twice with PBS containing 2% heat-inactivated FBS, and resuspended in PBS with 2% FBS at a concentration of 10^6^ cells per 100 μl. Antibodies at the appropriate dilution were added to the cells, and the mixture was incubated for 20 min on ice. Then, the sample was washed twice with PBS containing 2% FBS. The antibodies were anti-CD44 allophycocyanin (APC) (eBioscience, San Diego, CA, USA), anti-CD24 phycoerythrin (PE) (Beckman Coulter, Brea, CA, USA), anti-ESA-FITC (GeneTex, Irvine, CA, USA), and anti-CD44v, which was kindly provided by Dr Hideyuki Saya (Keio University, Tokyo, Japan). Flow cytometry analysis was performed by using a MACSQuant analyzer (Miltenyi Biotec, Gladbach, Germany), and results were analyzed with FlowJo software (TreeStar, OR, USA). CD24high/CD44high cells were then isolated and sorted from YPK-Lm by FACSAria III (BD Immunocytometry Systems, Franklin Lakes, NJ, USA). The sorted CD24high/CD44high cells were referred to as YPK2-SortLm and YPK5-SortLm.

### Analysis of ALDH activity

To assess the cellular ALDH activity, the Aldefluor assay kit (StemCell Technologies, Vancouver, BC, Canada) was used according to the manufacturer’s guidelines. Briefly, cells were harvested, placed in Aldefluor assay buffer (1×10^6^/ml), and incubated with Aldefluor substrate for 45 min at 37°C to allow substrate conversion. As a negative control for all experiments, an aliquot of Aldefluor-stained cells was immediately quenched with 1.5-mM diethylamino-benzaldehyde (DEAB), a specific ALDH inhibitor. Cells were analysed by using the green fluorescence channel (FL1) on a MACSQuant analyzer, and results were analyzed with FlowJo software. Cells that fell within the closed area were considered to represent subpopulations of cells with enhanced ALDH activity as compared with the rest of the cell population.

### Cell cycle phase distribution analysis

We performed the cell cycle analysis according to company recommendations (BD Bioscience, Franklin Lakes, NJ, USA). Briefly, cells were trypsinized and centrifuged at 1500 rpm for 5 min, washed twice with PBS, and then fixed with 70% cold ethanol. Fixed cells were stained by using PI/RNase Staining Buffer (BD Bioscience) and incubated for 15 min at room temperature before analysis. Analysis was performed with the MACSQuant analyzer, and results were analyzed with FlowJo software.

### Xenograft model

Rag^−/−^IL-2 common gamma chain^−/−^mice were purchased from the Jackson Laboratory (Bar Harbor, ME, USA) and bred and maintained in a HEPA-filtered environment with autoclave-sterilized cages, food, and bedding. All animal studies were conducted in accordance with the Institutional Animal Care and Use Committee of Yamaguchi University and conformed to the Guide for the Care and Use of Laboratory Animals published by the US National Institutes of Health. Mice were inoculated with 10^3^ or 10^4^ cells in each experiment. All mice were inoculated subcutaneously in the left lower abdominal quadrant with a 27-gauge needle.

### Semi-quantitative real-time RT-PCR

The expression levels of stemness genes (KIT, ALDH1A1, NANOG) and epithelial-mesenchymal transition (EMT)-related genes (CDH1, CDH2, VIM, FN1, SNAI1, SNAI2, ZEB1, ZEB2) were examined by RT-PCR. Semi-quantitative real-time RT-PCR was performed as described previously with minor modifications (15,16). RNAs were extracted from cells by using TRIzol reagent (Life Technologies). Reverse transcription was performed with the PrimeScript RT reagent kit (Takara Bio, Shiga, Japan). Real-time PCR amplification was performed by using LightCycler 480 Probe Master (Roche Diagnostics, Tokyo, Japan) and Universal ProbeLibrary probes (Roche Diagnostics) in a LightCycler System Version 3 (Roche Diagnostics). Primers and probes are listed in Table I. Amplification was performed according to a 2-step cycle procedure consisting of 45 cycles of denaturation at 95°C for 10 sec and annealing/elongation at 60°C for 30 sec. We measured mRNA levels semi-quantitatively by the Δ/Δ threshold cycle (Ct) method. Both the GAPDH and β-actin (ACTB) genes were used as reference genes. The values are expressed as relative to the parental cells.

### Measurements of cytokine and chemokine levels

Frozen aliquots of YPK2 and YPK5 were thawed and cultured for 2 weeks prior to harvesting culture supernatant from sub-confluent cultures (Sup-YPK2 and Sup-YPK5). YPK2-Lm and YPK5-Lm supernatant was harvested when cells were sub-confluent 1 month after transfer to laminin-coated dishes in the sphere culture medium (Sup-Lm2 and Sup-Lm5). The Bioplex assay (Bio-Rad, Marne la Coquette, France) was performed according to the manufacturer’s instructions to evaluate the levels of cytokines and chemokines in the supernatant. Samples were analyzed in triplicate. Experimental data were analyzed by using five-parametric curve fitting. We measured the protein level of the following 28 cytokines and chemokines: TGF-β, IL-1b, IL-1ra, IL-2, IL-4, IL-5, IL-6, IL-7, IL-8, IL-9, IL-10, IL-12, IL-13, IL-17, eotaxin, bFGF, G-CSF, GM-CSF, interferon (IFN)-γ, immune protein (IP)-10, monocyte chemotactic protein (MCP)-1, macrophage inflammatory proteins (MIP)-1α, MIP-1β, platelet-derived growth factor (PDGF)-BB, regulated on activation, normal T-cell expressed and secreted (RANTES), tumor necrosis factor (TNF)-α, and vascular endothelial growth factor (VEGF).

### Statistical analysis

The results are presented as means ± SD. Statistical differences were determined using the Mann-Whitney U tests. P-values of <0.05 were considered significant.

## Results

### Induction and culture of CSLC-enriched population

**Figure 1:**
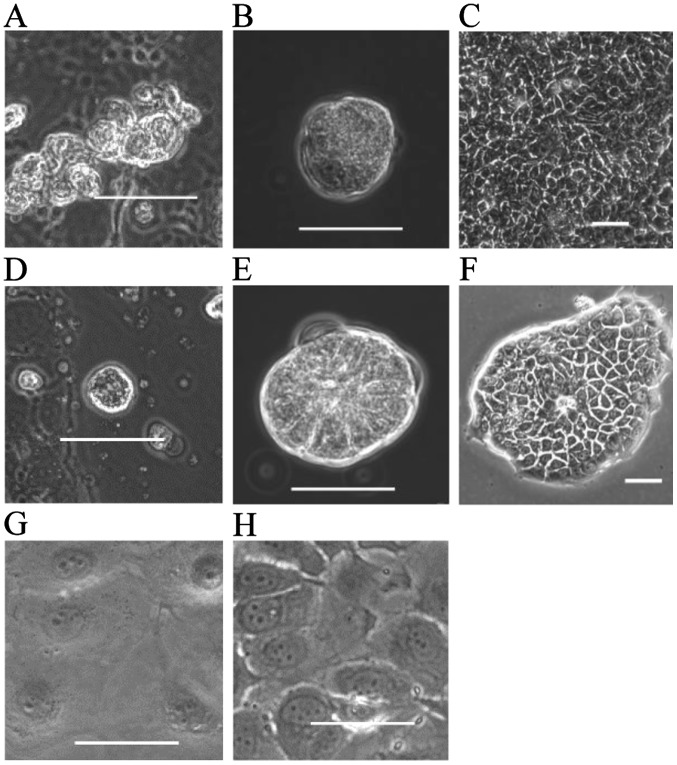
Sphere formation and induction of CSLC-enriched population. When YPK2 and YPK5 were initially cultured in the CSC-inducing media, a portion of cells formed spheres in suspension culture in a few hours [(A) YPK2; (D) YPK5]. These sphere cells were harvested on day 7 and transferred to laminin-coated dishes. (B) One week after YPK2-Sp was transferred to laminin-coated dishes. (C) Two months after YPK2-Sp was transferred to laminin-coated dishes. (E) One week after YPK5-Sp was transferred to laminin-coated dishes. (F) Two months after YPK5-Sp was transferred to laminin-coated dishes. YPK2 and YPK5 cultured in DMEM containing 10% FBS maintained the same morphology as parental cells [(G) YPK2; (H) YPK5]. Scale bars, 50 μm.

When YPK2 or YPK5 were initially cultured in the CSC-inducing media, cells began to attach on the plate, and a portion of cells formed spheres in suspension culture within a few hours (YPK2-Sp and YPK5-Sp) (Fig. 1A and D). These spheres grew to become larger sphere clusters within a week. YPK2-Sp or YPK5-Sp were harvested on day 7 and transferred to laminin-coated dishes. Cells began to attach to the dishes within a few hours; then, they gradually divided and the number of spheres and attached cells increased for 2 months (YPK2-Lm and YPK5-Lm, Fig. 1B, C, E and F). The surviving cells displayed both attached and cluster-formatted morphology. When these cells were grown in culture for >3 months, they became apoptotic without proliferation. Fig. 1G and H show YPK2 and YPK5 cultured in DMEM containing 10% FBS. These cells were attached and proliferated quickly.

### Cell surface markers

**Figure 2:**
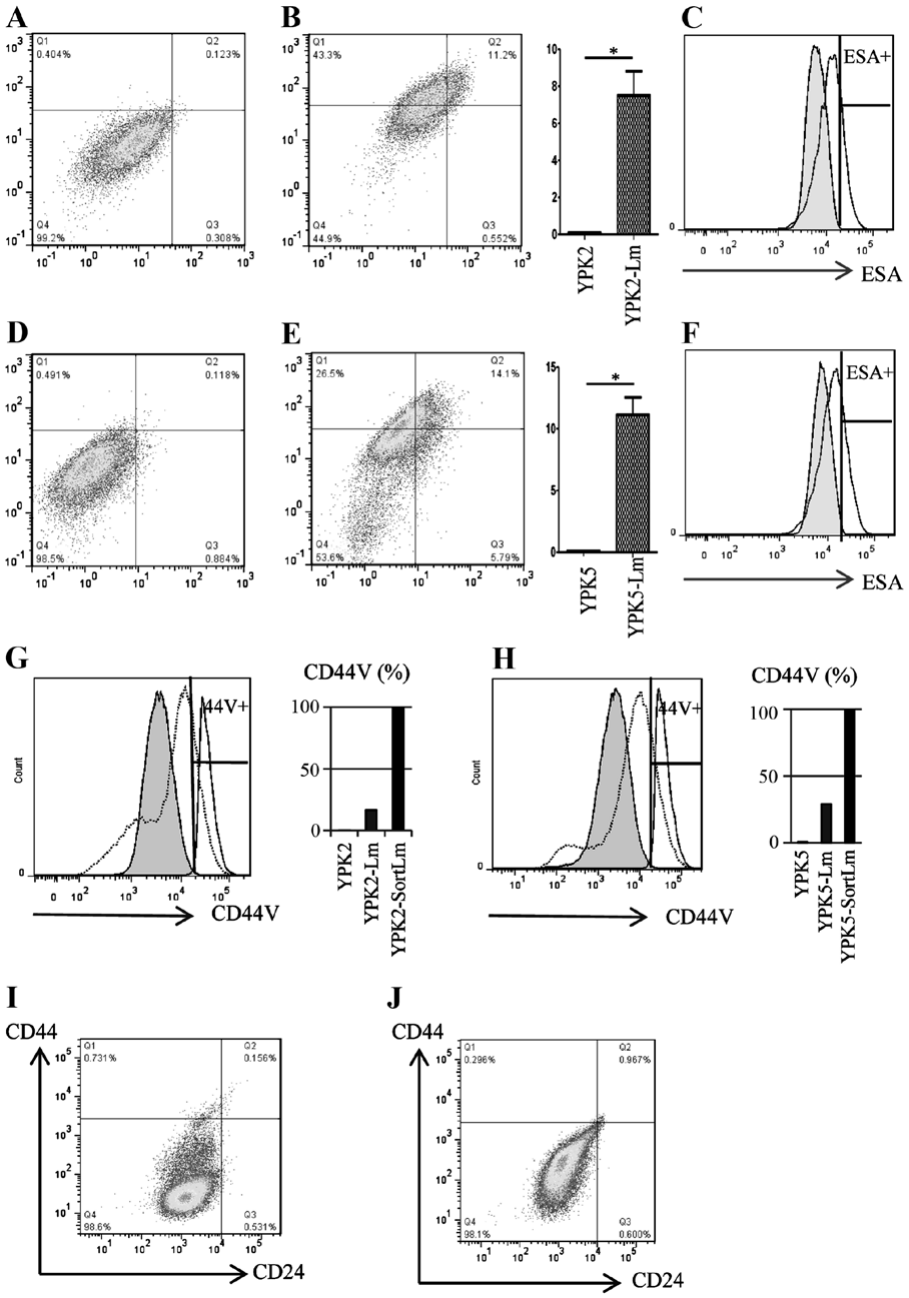
Expression of CD24, CD44, CD44v and ESA. The ratio of the expression of CD24high/CD44high in YPK2 and YPK5 was 0.1% [(A) YPK2; (D) YPK5]. The ratios of the expression of CD24high/CD44high in YPK2-Lm and YPK5-Lm were 7.5±2.6 and 11.1±2.8%, respectively [(B) YPK2-Lm; (E) YPK5-Lm]. Expression of ESA in YPK2-SortLm [(C) black line, 23.2%] and YPK5-SortLm [(F) black line, 36.2%] was higher than that of YPK2 [(C) tinted line, 0.1%] and YPK5 [(F) tinted line, 0.1%]. (G) The ratio of CD44v in YPK2 (tinted line) was only 0.2%, but the ratios in YPK2-Lm (dotted line) and YPK2-SortLm (black line) were increased to 16.7 and 99.8%, respectively. (H) The ratio of CD44v in YPK5 (tinted line) was only 0.2%, however these ratios of CD44v in YPK5-Lm (dotted line) and YPK5-SortLm (black line) were 16.7 and 99.8%, respectively. (I) YPK 2-Lm was cultured in the CSC-inducing media without NSF-1. (J) YPK 2-Lm was cultured in the CSC-inducing media without LIF. When NSF-1 or LIF were omitted from the CSC-inducing media, cells expressed cancer cell-like patterns of surface markers such as CD24low/CD44low. Incidences in (B) and (E) were evaluated by Mann-Whitney U tests; ^*^P<0.05.

In general, a CD44^+^/CD24^+^/ESA^+^ phenotype has stem-cell properties in pancreatic cancer cells (7). In the present study, the ratio of the expression of CD24high/CD44high in YPK2 (Fig. 2A) and YPK5 (Fig. 2D) was ~0.1%, while the ratios (mean ± SD) in YPK2-Lm (Fig. 2B) and YPK5-Lm (Fig. 2E) were significantly increased to 7.5±2.6% (P=0.0211) and 11.1±2.8% (P=0.0211), respectively. Expression of ESA in YPK2-SortLm (23.2%) and YPK5-SortLm (36.2%) was higher than that of YPK2 (0.1%) and YPK5 (0.1%) (Fig. 2C and F). Recently, some studies have focused on the role of CD44v in CSCs (17,18). We also focused on the expression of CD44v. Fig. 2G shows that YPK2-SortLm expressed a higher ratio of CD44v than YPK2-Lm and YPK2. The ratio of CD44v in YPK2 was only 0.2%; however, this ratio in YPK2-Lm and YPK2-SortLm was 16.7 and 99.8%, respectively. Expression of CD44v in YPK5-SortLm was also high compared to YPK5 (Fig. 2H). When NSF-1 or LIF was omitted from CSC-inducing media, CD24low/CD44low cells were dominant; these cells were also dominant in parent cancer cells (Fig. 2I and J).

### ALDH activity and cell cycle analysis

**Figure 3:**
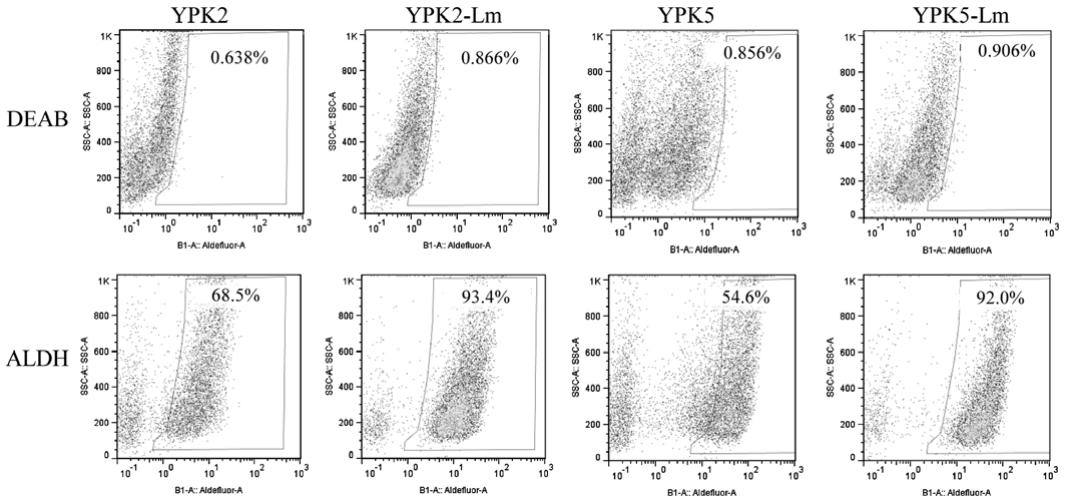
ALDH activity. The upper panels show representative dot plots of cells treated with the ALDH-specific inhibitor DEAB (negative controls). The lower panels show representative dot plots of ALDH activity. YPK2 and YPK5 expressed high levels of ALDH activity (YPK2, 68.5%; YPK5, 54.6%), however, YPK2-Lm and YPK5-Lm expressed much higher levels of ALDH activity (YPK2-Lm, 93.4%; YPK5-Lm, 92.0%) than parental cells.

A functional mechanism for chemo-resistance has been associated with ALDH activity (19). In the present study, YPK2 and YPK5 expressed high levels of ALDH activity (YPK2, 68.5%; YPK5, 54.5%), however, YPK2-Lm and YPK5-Lm expressed much higher levels of ALDH activity (YPK2-Lm, 93.4%; YPK5-Lm, 92.0%) than parental cells (Fig. 3).

**Figure 4:**
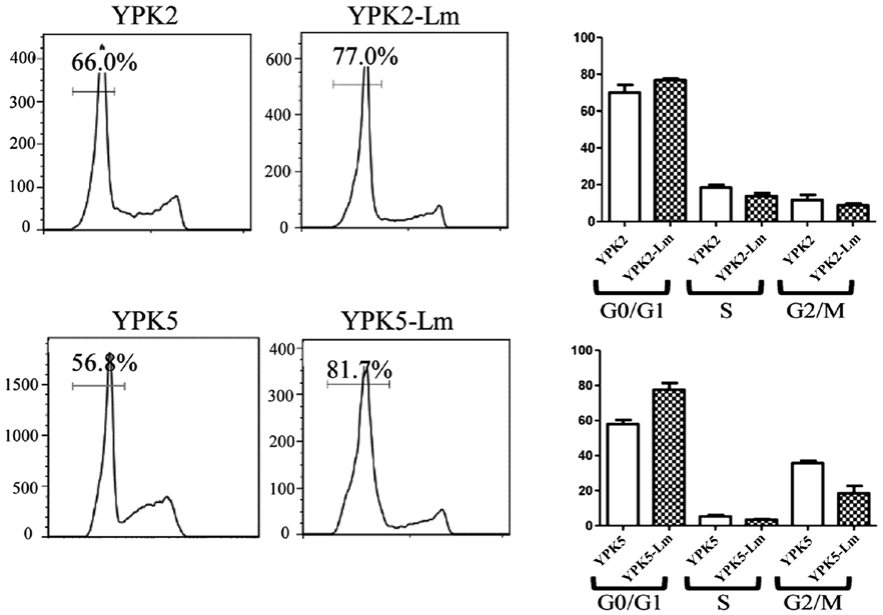
Cell cycle. Many of the YPK2-Lm and YPK5-Lm are relatively quiescent compared to the YPK2 and YPK5, however, these were not statistically significant. Evaluated by the Mann-Whitney U tests.

Stem cell quiescence is also highly relevant for chemotherapy against cancer, as it is retained and contributes to relapse following discontinuation of therapy (20). Many CSCs are non-cycling G0 cells and would not be susceptible to cell cycle-specific chemotherapy agents. Many of YPK2-Lm and YPK5-Lm are relatively quiescent compared to the YPK2 and YPK5, however, these were not statistically significant (Fig. 4).

### Tumorigenicity

YPK2-SortLm cells gave rise to new tumors in 3 of 3 mice, ≥10^3^ cells were injected. In contrast, no tumors formed when 10^3^ YPK2 cells were injected, which demonstrates the much higher tumorigenicity of YPK2-SortLm cells.

### mRNA expression of stem-cell and mesenchymal markers

**Figure 5:**
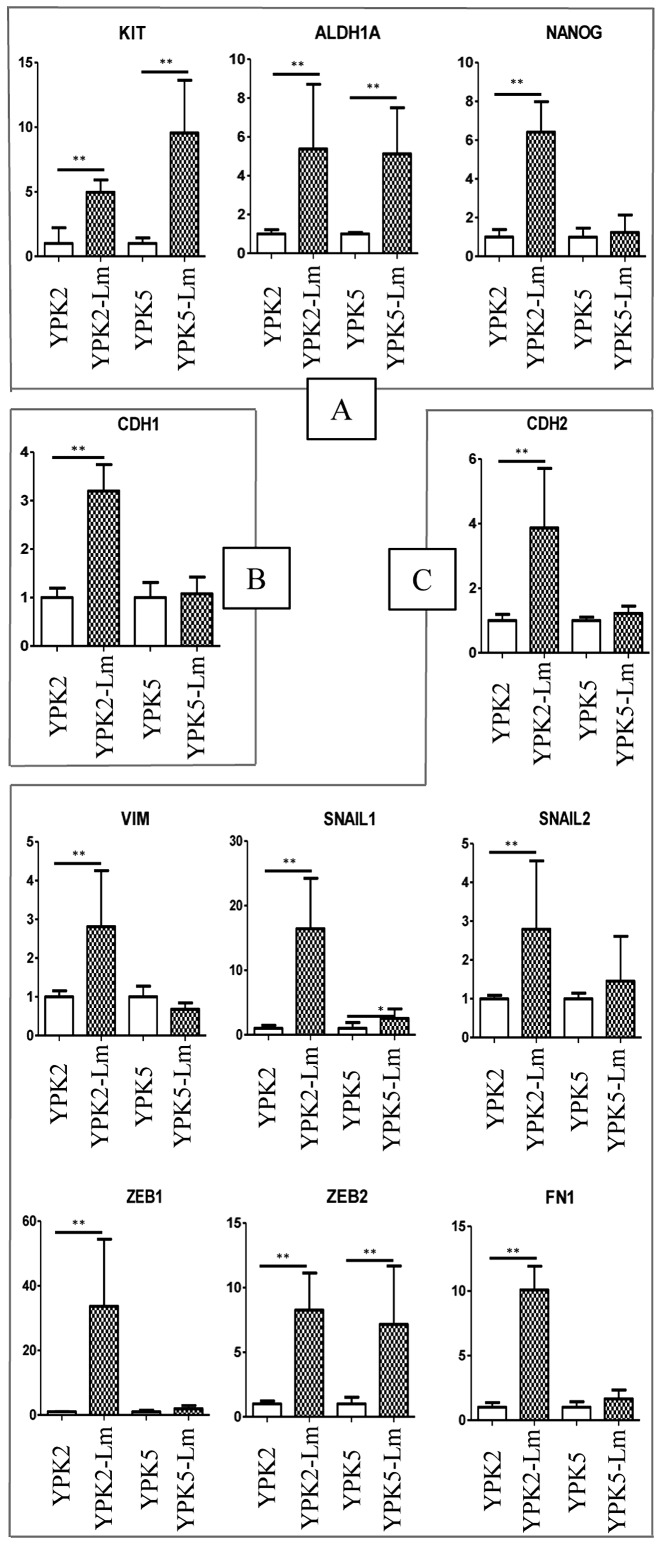
mRNA expression of stemness and EMT-related markers. (A) The expression levels of stemness genes. RT-PCR resulted in significantly higher expression levels of KIT and ALDH1A1 in both YPK2-Lm (P=0.0095 and 0.0022, respectively) and YPK5-Lm (P=0.0022 and 0.0049, respectively). (B) The expression levels of epithelial genes. The expression level of CDH1 was significantly higher in YPK2-Lm than YPK2, but was not statistically significant between YPK5 and YPK5-Lm. (C) The expression levels of mesenchymal genes. The expression levels of CDH2, VIM, SNAI1, SNAI2, ZEB1, ZEB2, and FN1 were significantly higher in YPK2-Lm than in YPK2 (P=0.0022, respectively). In YPK5-Lm, the expression levels of SNAI1 and ZEB2 were significantly higher than in YPK5 (P=0.026 and 0.0087, respectively). Evaluated by the Mann-Whitney U tests; ^*^P<0.05; ^**^P<0.01.

The theory of the relationship between EMT and CSCs has been supported recently by the fact that cancer cells with migratory and invasive capabilities associated with metastatic competence are caused through EMT (21–23). Recent studies have established a crucial link between passage through EMT and the acquisition of the molecular and functional properties of stem cells (24,25). We therefore confirmed whether YPK-Lm have EMT properties (Fig. 5). RT-PCR resulted in significantly higher expression levels of stemness genes such as KIT and ALDH1A1 in both YPK2-Lm (P=0.0095 and 0.0022, respectively) and YPK5-Lm (P=0.0022 and 0.0049, respectively) (Fig. 5A). The expression level of NANOG was significantly high in only YPK2-Lm (YPK2-Lm; P=0.005, YPK-5Lm; P=0.9361). The expression levels of mesenchymal genes such as CDH2, VIM, SNAI1, SNAI2, ZEB1, ZEB2, and FN1 were significantly higher in YPK2-Lm than in YPK2 (P=0.0022, respectively) (Fig. 5C). In YPK5-Lm, the expression levels of SNAI1 and ZEB2 were significantly higher than in YPK5 (P=0.026 and 0.0087, respectively). The expression level of CDH1 was significantly higher in YPK2-Lm than YPK2, but was not statistically significant between YPK5 and YPK5-Lm (Fig. 5B).

### Cytokine analysis in the culture media

**Figure 6:**
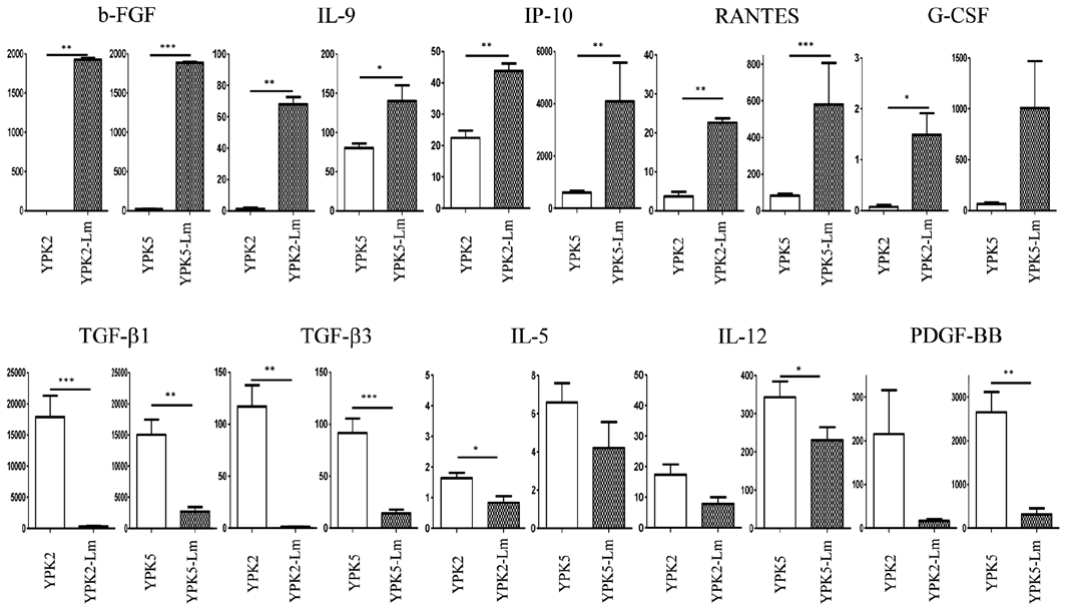
Cytokine assays of culture media. The levels of b-FGF, IL-9, IP-10, and RANTES were significantly detected as higher concentrations in the Sup-Lm2 and Sup-Lm5 compared to the Sup-YPK2 and Sup-YPK5 (P<0.05). The levels of G-CSF was also significantly detected as higher concentrations in the Sup-Lm2 compared to the Sup-YPK2 (P=0.02), and also higher trend in the Sup-Lm5 than in the Sup-YPK5 (P=0.06). The levels of TGF-β1 and TGF-β3 were detected as higher concentrations in the Sup-YPK2 and Sup-YPK5 compared to the Sup-Lm2 and Sup-Lm5 (P<0.01). The level of IL-5 was significantly detected as higher concentrations in the Sup-YPK2 compared to the Sup-Lm2 (P=0.015), and also higher trend in the Sup-YPK5 than in the Sup-Lm5 (P=0.07). The levels of IL-12 and PDGF-BB were also significantly detected as higher concentrations in the Sup-YPK5 compared to the Sup-Lm5 (P=0.04, and 0.0027), although these were not statistically significant between the Sup-YPK2 and Sup-Lm2 (P=0.0939 and 0.0926). Evaluated by the Mann-Whitney U tests; ^*^P<0.05; ^**^P<0.01; ^***^P<0.001.

To question the interaction of microenvironment between cancer and CSLC, we performed multiple cytokine assays with their culture media (Fig. 6). The levels of b-FGF, IL-9, IP-10, and RANTES were significantly detected as higher concentrations in the Sup-Lm2 and Sup-Lm5 compared to the Sup-YPK2 and Sup-YPK5 (P<0.05). The level of G-CSF was also significantly detected as higher concentrations in the Sup-Lm2 compared to the Sup-YPK2 (P=0.02), and also higher trend in Sup-Lm5 than in Sup-YPK5 (P=0.06). The levels of TGF-β1 and TGF-β3 were detected as higher concentrations in the Sup-YPK2 and Sup-YPK5 compared to the Sup-Lm2 and Sup-Lm5 (P<0.01). The level of IL-5 was significantly detected as higher concentrations in the Sup-YPK2 compared to the Sup-Lm2 (P=0.015), and also higher trend in the Sup-YPK5 than in the Sup-Lm5 (P=0.07). The levels of IL-12 and PDGF-BB were also significantly detected as higher concentrations in the Sup-YPK5 compared to the Sup-Lm5 (P=0.04 and 0.0027), although these were not statistically significant between the Sup-YPK2 and Sup-Lm2 (P=0.0939 and 0.0926).

## Discussion

We established a novel culture method to induce a P-CSLC-enriched population from human pancreatic cancer cell lines. As a first step, human pancreatic cancer cell lines were cultured and induced to form spheres/aggregates within a week. As a second step, these sphere cells were transferred to a laminin-coated dish with the medium, attached and the population of these induced cells expanded within a few months. In the present study, the ratio of CD24high/CD44high cells in YPK-Lm was enriched (Fig. 2B and E). Almost all of YPK-Lm expressed CD44v and also expressed high levels of ALDH activity (Figs. 2G and 3). Cell-cycle analysis showed that many YPK-Lm preferentially stayed in the G0/G1 phase (Fig. 4). mRNA levels of mesenchymal markers such as SNAI1 and ZEB2 were expressed in YPK-Lm as we expected (Fig. 5C). Similarly, RT-PCR resulted in higher expression levels of stemness marker such as KIT and ALDH1A1 from YPK-Lm (Fig. 5A). These results suggest that YPK-Lm acquired stemness properties through the EMT. The expression level of CDH1, which is an epithelium-related gene expected to be high in YPK based on this EMT theory, was high in YPK-Lm (Fig. 5B). Thus, this theory of CSC induction by passage through the EMT still has room for argument. Based on the facts described above, we confirmed that these induced cells have CSCs characteristics.

A prominent feature of CSCs is their ability to form floating spheroids in serum-free culture conditions (26). Several studies have suggested that CSCs can be enriched in spheres when cultured in serum-free medium supplemented with adequate mitogens, such as bFGF and EGF (27–30). However, culture cells kept in the sphere formation for >10 days forfeit not only their stemness properties but also viability. The most problematic issue is the spontaneous differentiation and cell death that accompany stem cell divisions in the sphere environment (31). In contrast, most individual cells in adherent culture conditions are uniformly exposed to defined growth factors and oxygen tension, which allows most CSCs to maintain their stemness properties without spontaneous differentiation and cell death. The laminins are an important and biologically active part of the basal lamina, influencing cell differentiation, migration, and adhesion, as well as survival (32,33). To overcome the limitations of the neurosphere culture paradigm, Pollard *et al* cultured glioma tumor-initiating cells as adherent cell lines by using laminin-coated dishes (31). In our experiment, the modified stem cell medium with NSF-1, and LIF induced a P-CSLC-enriched population, however, the medium without NSF-1 and/or LIF failed to induce this population (Fig. 2I, and J). In addition, this induced population did not divide and the number of cells did not increase in this condition without transferring to laminin-coated dishes. This population has to be transferred to laminin-coated dishes approximately one week after sphere formation. Then, this population is able to maintain the stemness properties and viability with self-renewing properties. We suggest that the process of CSLC induction demands the neural stimulation factors with some adequate cytokines and chemokines, such as bFGF and EGF. Based on our data of cytokines from the supernatant, it was established that induced and maintained conditions between CSCs and cancer cells are drastically different in terms of cytokines profile in the culture (Fig. 6). As typical examples, b-FGF, IL-9, IP-10, RANTES, and G-CSF were higher in supernatant of CSCs culturing, while TGF-β1, TGF-β3, IL-5, IL-12, and PDGF-BB were higher in supernatant of cancer cells culturing. Needless to say, this part of the study is immature and weak. Further analysis and study will be required to reveal the mechanism inducing CSLCs in the culture.

Currently, CSC-targeting therapy has been attempted to be established (34,35), because conventional anticancer treatments do not target CSCs and have no efficacy against CSCs. However, one of the difficulties in the quest to characterize the CSC population from tumor specimens is the rarity of this population. Using the method as established in this study, we can easily enrich the CSLC population without special instruments. Although this method is potentially able to be applied to freshly harvested cancer tissue, further investigations in this area are needed. We are planning to use these induced cells to establish a novel immunotherapy targeting CSCs through proteomics. For screening the ability of the immune effector cells to eradicate their target-CSCs, an appropriate number of CSCs can be used with this novel technology.

In conclusion, we established a culture method to induce a CSLC-enriched population from human pancreatic cancer cell lines. This method may be useful to analyze CSC characteristics in detail, and to help in the establishment of novel therapies against CSCs.

## Figures and Tables

**Table I:** Primers and probes.

Symbol	Name	UPL probe no.	Sequence (5′-3′)
KIT (C-Kit, CD117)	KIT-S	71	ctttcctcgcctccaagaat
	KIT-AS		gtgatccgaccatgagtaagg
ALDH1A1	ALDH1A1-S	14	tttggtggattcaagatgtctg
	ALDH1A1-AS		cactgtgactgttttgacctctg
NANOG	NANOG-S	31	agatgcctcacacggagact
	NANOG-AS		tttgcgacactcttctctgc
CDH1 (E-cadherin)	CDH1-S	35	cccgggacaacgtttattac
	CDH1-AS		gctggctcaagtcaaagtcc
CDH2 (N-cadherin)	CDH2-S	80	agtatccggtccgatctgc
	CDH2-AS		ctgtggggtcattgtcagc
VIM (vimentin)	VIM-S	13	tacaggaagctgctggaagg
	VIM-AS		accagagggagtgaatccag
FN1	FN1-S	60	aagagcgagcccctgatt
	FN1-AS		atgaagattggggtgtggaa
SNAI1	SNAI1-S	10	catgtccggacccacact
	SNAI1-AS		tggcactggtacttcttgaca
SNAI2 (SLUG)	SNAI2-S	7	tggttgcttcaaggacacat
	SNAI2-AS		gttgcagtgagggcaagaa
ZEB1	ZEB1-S	36	cctaaaagagcacttaagaattcacag
	ZEB1-AS		catttcttactgcttatgtgtgagc
ZEB2 (SIP1)	ZEB2-S	68	aagccagggacagatcagc
	ZEB2-AS		ccacactctgtgcatttgaact
